# Genome-Wide Identification and Expression Analysis of Respiratory Burst Oxidase Homolog (*RBOH*) Gene Family in Eggplant (*Solanum melongena* L.) under Abiotic and Biotic Stress

**DOI:** 10.3390/genes14091665

**Published:** 2023-08-23

**Authors:** Lihui Du, Zheng Jiang, Yadong Zhou, Lei Shen, Jie He, Xin Xia, Longhao Zhang, Xu Yang

**Affiliations:** College of Horticulture and Landscape Architecture, Yangzhou University, Yangzhou 225009, Chinajie.he@yzu.edu.cn (J.H.); mz120221425@stu.yzu.edu.cn (X.X.); mz120221438@stu.yzu.edu.cn (L.Z.)

**Keywords:** eggplant, *RBOHs*, abiotic stress, *V. dahliae*, subcellular localization

## Abstract

Respiratory burst oxidase homologs (*RBOHs*) are important proteins that catalyze the production of reactive oxygen species (ROS), which play important roles in growth and stress response. For a comprehensive analysis of *SmRBOH* genes, we conducted genome-wide identification of the *SmRBOH* gene family in eggplant (*Solanum melongena* L.) and analyzed the expression of *SmRBOHs* under abiotic (salt, high-temperature, and low-temperature) and biotic stress (*Verticillium dahliae* inoculation) by quantitative real-time PCR (qRT-PCR). The result showed that a total of eight *SmRBOH* members were identified from the genome database of eggplant, and they were relatively evenly distributed across seven chromosomes. The analysis of Motif and the conserved domain showed that *SmRBOHs* have high similarity in protein sequences and functions. Based on phylogenetics, *SmRBOHs* were classified into three distinct clades. Furthermore, the promoter regions of *SmRBOHs* were found to contain different cis-elements. Additionally, the results of the qRT-PCR demonstrated differential expression patterns of *SmRBOHs* in different tissues (the roots, stems, and leaves) and stress conditions. *SmRBOHB*, *SmRBOHD*, *SmRBOHH1*, and *SmRBOHH2* showed significant upregulation (>20-fold) under at least one stress condition. Subcellular localization analysis of the above four members further confirmed that they localized on the plasma membrane. This study provides a theoretical foundation for understanding the functions of SmRBOHs in eggplant.

## 1. Introduction

Reactive oxygen species (ROS) are signaling molecules that include singlet oxygen (^1^O_2_), superoxide anion (O_2_^−^), hydrogen peroxide (H_2_O_2_), and a hydroxyl radical (HO^−^). Plants produce low concentrations of ROS to maintain normal growth and metabolism under normal conditions. However, plants generate a large amount of ROS under stress conditions. On the one hand, the accumulation of ROS can cause oxidative damage to the plants themselves. On the other hand, under stress conditions, ROS, especially H_2_O_2_, act as signaling molecules and participate in the process of programmed cell death [1,2]. Nicotinamide adenine dinucleotide phosphate (NADPH) oxidases (*NOXs*), also known as respiratory burst oxidase homologs (*RBOHs*), are the key enzymes responsible for the generation of ROS [3]. *RBOHs* are predominantly localized to the plasma membrane and function as part of the plasma membrane redox system, transferring electrons from intracellular NADPH/NADH to oxygen, leading to the production of a large amount of O^2−^. Subsequently, the O^2−^ is disproportionated to produce H_2_O_2_ and other ROS [4]. In animals, *NOXs* consist of six subunits, with gp91^phox^ being the central functional subunit. Based on sequence alignment with gp91^phox^, multiple *RBOHs* have been identified in plants [5]. The C-terminal region of RBOHs exhibits a high similarity to human gp91^phox^, possessing conserved domains such as the FAD and NADPH binding domains. Unlike gp91^phox^, the N-terminal of plant *RBOHs* typically contains two EF-hand Motifs associated with Ca^2+^ binding and phosphorylation sites, indicating the potential regulation of *RBOH* by Ca^2+^ binding and phosphorylation [6]. The first *RBOH* gene in plants was identified from rice (*Oryza sativa* L.), and subsequently, multiple *RBOH* genes have been cloned from other plants such as tomato (*Solanum lycopersicum* L.), potato (*Solanum tuberosum* L.), and tobacco (*Nicotiana tabacum* L.) [7,8,9]. The *RBOH*-dependent ROS burst can be effectively inhibited by the specific inhibitor of the animal NADPH oxidase, diphenyleneiodonium (DPI), suggesting functional similarities between plant *RBOHs* and animal gp91^phox^ [10]. In addition to higher plants, *RBOH* genes have also been identified in algae, and compared to higher plants, *RBOHs* in diatoms *Thalassiosira pseudonana* and the brown alga *Phaeodactylum tricornutum* are more closely related to animal gp91^phox^ [11,12], indicating that *RBOHs* are ancient genes that appeared in eukaryotic unicellular organisms as well.

The *RBOH* gene family has also been identified in several species. Members of this family have different physiological functions, which are important in stress response, growth and metabolism, and phytohormone regulation [13,14,15]. The ROS generated by RBOH proteins have profound effects on plant morphogenesis. For instance, *AtRBOHC* is involved in root hair growth in *A. thaliana* (*Arabidopsis thaliana* L.), while *AtRBOHD* and *AtRBOHF* lead to the local accumulation of superoxides in the roots of *A. thaliana*, thereby inhibiting lateral root development [16]. Moreover, *OsRBOHA* participates in the germination process of rice seeds and pollen, and its knockout results in reduced pollen viability and seed fertility [17].

In the face of abiotic stress, RBOH-dependent ROS are speculated to serve as signaling molecules, initiating cascade reactions that activate various defense mechanisms. Among the seven *RBOH* genes in citrus (*Citrus reticulata Blanco*), five *CsRBOH* genes have been found to respond to cold stress, and the knockout of *CsRBOHD* reduces plant cold tolerance [18]. Furthermore, studies have confirmed the involvement of *RBOHs* in the maintenance process of cold acclimation in cucumber (*Cucumis sativus*) [19]. H_2_O_2_ generated by *AtRBOHD* and *AtRBOHF* promotes the influx of Ca^2+^, a crucial step in signal transduction, which to some extent, maintains the Na^+^/K^+^ balance under salt stress. The double mutant of *AtRBOHD* and *AtRBOHF* shows significant inhibition of Ca^2+^ influx under salt stress, resulting in decreased salt tolerance in *A. thaliana* [20]. In rice, drought stress induces the activity of RBOH oxidases. Overexpression of *OsRBOHA* and *OsRBOHB* enhances plant drought tolerance. *OsRBOHB* is mainly involved in ROS production and abscisic acid (ABA) signal transduction. Mutants of *OsRBOHB* not only show reduced ROS production but also exhibit lower ABA content and larger stomatal apertures, leading to decreased drought resistance [21]. Under biotic stress conditions, RBOH-dependent ROS participate in plant immune responses, reinforcing cell walls, enhancing defense against pathogens, and playing a role in signal transduction, thereby increasing plant resistance to pathogens [22,23,24]. Verticillium wilt is a soil-borne disease primarily caused by *V. dahliae* (*Verticillium dahlia*). Virus-induced gene silencing (VIGS) and overexpression confirmed that *GbRBOH5/18* was involved in the resistance of cotton to verticillium wilt [22].

Eggplant (*S. melongena* L.) is an important vegetable crop with rich nutrition and significant economic value. However, its growth cycle is lengthy, and its yield is often influenced by various adverse environmental factors [25]. Although numerous studies have demonstrated the regulatory roles of *RBOHs* in plant growth, development, and responses to stress [14,15,26], there have been no reports on *SmRBOHs*. In this study, we identified and conducted a bioinformatic analysis of the *SmRBOH* gene family in eggplant. We also analyzed the expression patterns of *SmRBOHs* in different tissues of eggplant and investigated their expression profiles under simulated salt, high-temperature and low-temperature stresses, and *V. dahliae* inoculation in eggplant seedlings. Additionally, we performed subcellular localization analysis for *SmRBOH* members that showed significant upregulation (>20-fold) under different stress treatments. This study aims to analyze the physicochemical properties, chromosome localization, Motif and conserved structure, phylogenetic evolutionary, cis-acting elements, the expression of *SmRBOHs* gene family, and identify *SmRBOHs* that play an important role under stress according to their expression under different stresses, providing a basis for subsequent research and utilization of *SmRBOH* genes.

## 2. Materials and Methods

### 2.1. Identification, Physicochemical Characterization, and Chromosomal Localization of SmRBOHs

In order to obtain the eggplant protein sequences, the genome data were downloaded from the eggplant Genome Database (http://eggplant-hq.cn/ accessed on 17 February 2023). TBtools v 1.120 [27] was utilized to extract the eggplant protein sequences. These sequences were then aligned with the already identified amino acid sequences of *A. thaliana RBOHs* through a double alignment [7]. To validate the alignments and identify conserved domains, further verification was conducted using the National Center for Biotechnology Information (NCBI) database (https://www.ncbi.nlm.nih.gov/ accessed on 12 March 2023). Subsequently, the final eggplant *RBOH* gene family members were identified and named. The physicochemical properties of the *SmRBOHs* were analyzed on Expasy (https://web.expasy.org/protparam/ accessed on 12 March 2023), which provided information on the isoelectric point, instability index, hydrophilicity, and other relevant properties. Additionally, the subcellular localization of *SmRBOH* family members was predicted using WOLFSORT (https://wolfpsort.hgc.jp/ accessed on 12 March 2023). Chromosome localization analysis and visualization of the *SmRBOHs* were performed using the eggplant genome annotation files and the Tbtools [27].

### 2.2. Motif and Conserved Domain Analysis of the SmRBOHs Family

Using the amino acid sequences of *SmRBOHs*, we analyzed the Motifs of *SmRBOHs* in the MEME Suite v 5.5.2 (https://meme-suite.org/ accessed on 18 April 2023) website. We performed the analysis of conserved domains in the *SmRBOH* family using the CD (Conserved Domain) Search available on NCBI and visualized the results.

### 2.3. Phylogenetic Analysis of SmRBOHs

According to previous research, we obtained the protein sequences of *SlRBOHs*, *AtRBOHs*, and *OsRBOHs* in tomato, Arabidopsis, and rice [7,8]. Based on the *SlRBOHs*, *AtRBOHs*, and *OsRBOHs* protein sequences, combined with the identified *SmRBOH* proteins, a multiple sequence alignment was performed using the ClustalW method of MEGA 11 (detailed information in Tables S1 and S2), and we constructed a phylogenetic evolutionary tree using the Neighbor-Joining (NJ) method with the Bootstrap method set to 1000 replications. Additionally, we used Evolview (http://www.evolgenius.info/evolview/#/ accessed on 30 April 2023) to visualize the phylogenetic tree.

### 2.4. Analysis of SmRBOHs Cis-Acting Elements in Plants

To predict the potential cis-acting element, the genomic DNA sequences 2000 bp upstream of the initiation codons of *SmRBOHs* were extracted Using TBtools [27]. Then we used PlantCARE (http://bioinformatics.psb.ugent.be/webtools/plantcare/html/ accessed on 12 May 2023) to analyze the putative cis-regulatory elements of *SmRBOHs* and used TBtools to visualize the results.

### 2.5. Plant Materials and Treatments

In this study, the materials were the inbred eggplant variety “JS221,” which had been self-pollinated for several years. Ripe eggplant seeds were enclosed in gauze, soaked in water at 55 °C for 15 min, and then removed and kept moist. Once the seed embryos exposed their radicles, they were transferred to 7 × 7 small pots filled with nutrient soil, with one seedling per pot. The pots were then placed in a controlled growth chamber (Lishigao Instrument Equipment Co., Ltd., Nanjing, China) with a photoperiod of 16 h light/8 h dark, a temperature of 25 °C/18 °C, light intensity of 800 µmol m^−2^ s^−1^, and relative humidity of 70%. When the seedlings reached the stage of 4–6 true leaves, four healthy eggplant seedlings were selected for analyzing the relative expression patterns of *SmRBOHs* in different tissues. Roots, stems, and leaves were collected, flash-frozen in liquid nitrogen, and stored at −80 °C for later analysis. To simulate salt stress, a 150 mmol/L NaCl solution was prepared and applied by watering each pot with 50 mL of the NaCl solution, with a tray placed under the pots to prevent salt leakage [28]. For the heat stress treatment, eggplant seedlings were exposed to a high temperature of 42 °C, while other conditions remained the same. To induce low-temperature stress, eggplant seedlings at the 4–6 true leaf stage were placed at 4 °C while other conditions remained constant. The treatment with *V. dahliae* involved watering the soil around the roots of the eggplant with a suspension of *V. dahliae* “*VdLS17*” spores, with a concentration of 10^7^ spores/mL, and 20 mL of the spore suspension was added to each pot [29,30]. Samples were then collected at different time points (0, 3, and 6 h) for each treatment. For NaCl and *V. dahliae* treatments, the root samples of eggplants were collected, while for temperature stress treatment, leaf samples were collected. Four randomly selected seedlings were used as biological replicates for each treatment.

### 2.6. qRT-PCR Assay

The cDNA preparation method and reagents used were referenced from previous research reports [28,31]. ChamQ SYBR qPCR Master Mix (Q311, Novogene, Nanjing, China) was utilized for qRT-PCR with SmActin as the reference gene. The qRT-PCR program and reaction system were consistent with those described in previous research reports [28,31]. The primer sequences are provided in Table S3.

### 2.7. Statistical Calculations

We used the 2^−ΔΔCt^ method to calculate relative expression levels and used a one-way analysis of variance and Tukey’s test (*p* < 0.01) to analyze the differences between samples. The statistical calculations were performed using Microsoft Office Excel 2021 and IBM SPSS Statistics v 22, and the graphical representation was processed using GraphPad Prism v 9.5.0. [31].

### 2.8. Subcellular Localization

The vector used in this experiment is pBinGFP2, and the material used is *N. benthamiana* (*Nicotiana benthamiana* L.). We selected significantly upregulated members of *SmRBOHs* (*SmRBOHB*, *SmRBOHD*, *SmRBOHE1*, *SmRBOHH2*) under at least one stress condition. Following the methods established by previous researchers [28,31], *SmRBOHs* were cloned and inserted into the pBinGFP2 vector. The constructed vectors, along with the empty pBinGFP2 vector (used as a control), were transformed into *Agrobacterium tumefaciens* strain GV3101. The *Agrobacterium* containing the desired vectors was adjusted to an OD_600_ of 0.8 using the infection solution, and the formula of the infection solution is reported in previous reports [28,31]. The OD_600_ = 0.8 suspension of *Agrobacterium* was aseptically injected into the leaves of *N. benthamiana* using 1 mL syringes. After 48 h, the fluorescence signals in the leaves were observed using a laser scanning confocal microscope (LSM 880NLO; Leica Microsystems, Wetzlar, Germany). The primer sequences required for the cloning of *SmRBOHs* are provided in Table S4

## 3. Results

### 3.1. Identification, Physicochemical Characterization, and Chromosomal Localization of SmRBOHs

**Figure 1:**
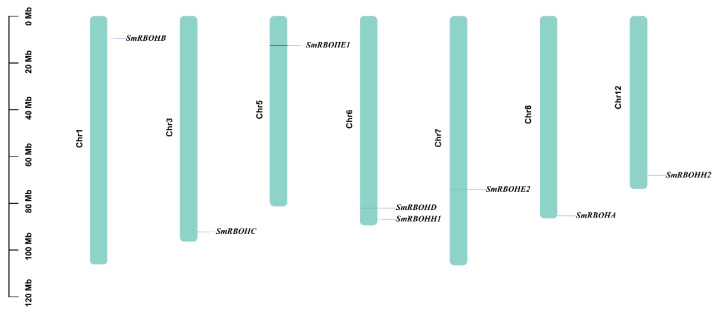
Chromosomal localization of the *SmRBOH* gene family members. Bars represent the length of each chromosome displayed in megabases (Mb) on the left side. The labeled positions indicate the locations of SmRBOH genes on the chromosomes.

Using bioinformatics methods, eight *SmRBOH* genes were identified from the genome database of eggplant. These eight *SmRBOH* genes are relatively evenly distributed across seven chromosomes. Among them, chromosome 6 contains two members, namely *SmRBOHD* and *SmRBOHH1*. Additionally, chromosomes 1, 3, 5, 7, 8, and 12 each harbor one *SmRBOH* member (Figure 1).

The length of *SmRBOH* proteins ranges from 718 (*SmRBOHD*) to 963 amino acids (*SmRBOHA*), with relative molecular weights ranging from 82.136 to 109.25 KDa, suggesting potential structural differences. The isoelectric points are 8.72 (*SmRBOHE1*) to 9.41 (*SmRBOHD*), all of which are greater than 7.00, indicating an alkaline nature. *SmRBOHC*, *SmRBOHD*, and *SmRBOHH2* exhibit instability coefficients ranging from 37.44 to 39.24, indicating stable proteins, while the rest have coefficients ranging from 41 to 49.15, indicating unstable proteins. Analysis of the hydrophilicity of *SmRBOH* revealed negative values, indicating that all *SmRBOHs* are hydrophilic proteins but with varying degrees of hydrophilicity. Subcellular localization prediction analysis suggests that all eight members of the *SmRBOH* gene family are most likely located on the plasma membrane. These results indicate that *SmRBOHs* share certain physicochemical properties, such as being alkaline, hydrophilic proteins, and most likely localized on the plasma membrane (Table 1).

### 3.2. Motif and Conserved Structural Domain Analysis of the SmRBOH Family

**Figure 2:**
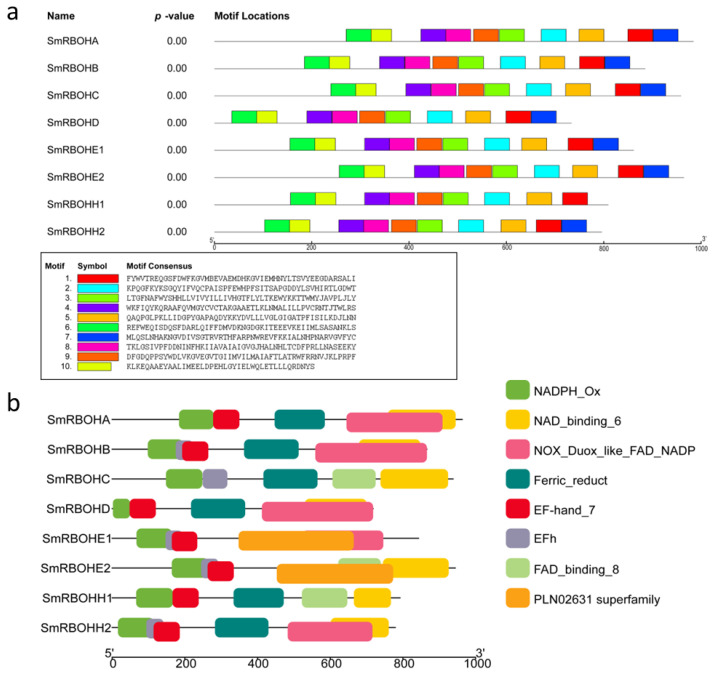
Motif and conserved domain analysis of *SmRBOHs*. The different lines represent protein lengths in amino acid (aa) units. (**a**) Distribution of 10 Motifs in *SmRBOHs*. Different colored rectangles represent distinct Motifs, with the annotations below the figure indicating the sequences of each Motif. (**b**) Distribution of conserved domains on *SmRBOH* proteins. Different colored blocks represent the presence of conserved domains on the *SmRBOH* proteins.

This study utilized the protein sequences of *SmRBOHs* to predict 10 Motifs (Figure 2a). Except for Motif 7, which is absent in *SmRBOHH1*, the other members contain all the Motifs, and the arrangement of the Motifs is entirely consistent. This indicates a high conservation of protein sequences among *SmRBOH* family members, suggesting potential functional similarities. The conserved domains analysis of *SmRBOH* revealed multiple conserved domains in this gene family (Figure 2b). All *SmRBOHs* have the conserved domain NADPH_Ox at the 5′(N-) terminal, and except for *SmRBOHE1*, other *SmRBOH* members have the NAD-binding domain NAD_binding_6 at the 3′(C-) terminal. With the exception of *SmRBOHC*, other *SmRBOH* members contain EF-hand_7, and except for *SmRBOHA*, *SmRBOHD*, and *SmRBOHH1*, the other *SmRBOH* members contain EFh. Both EF-hand_7 and EFh are recognized as EF-hand conserved domains associated with calcium ion binding. Except for *SmRBOHE1* and *SmRBOHE2*, others contain Ferric_reduct, while *SmRBOHE1* and *SmRBOHE2* possess the PLN02631 superfamily; Ferric_reduct and the PLN02631 superfamily are related to ferric reductase. *SmRBOHC*, *SmRBOHE2*, and *SmRBOHH1* contain FAD_binding_8, whereas other family members at this position have NOX_Duox_like_FAD_NADP. Both FAD_binding_8 and NOX_Duox_like_FAD_NADP are Ferredoxin reductase (FNR) domains involved in the photosystem electron transfer process [32,33,34].

### 3.3. Phylogenetic and Evolutionary Analysis of RBOHs

**Figure 3:**
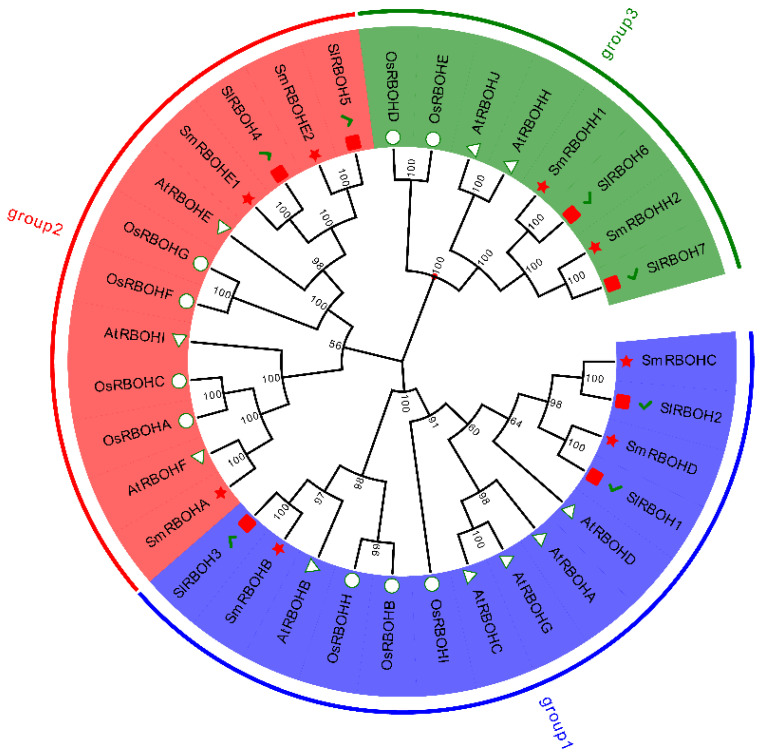
Phylogenetic and evolutionary analysis of *RBOHs* of eggplant, tomato, Arabidopsis, and rice. Amino acid sequences of *RBOH* genes from eggplant, tomato, Arabidopsis, and rice were aligned using MEGA 11. The phylogenetic tree was constructed using the Neighbor-Joining (NJ) method, and Bootstrap values were set to 1000. The analysis involved 34 *RBOH* genes. Eggplant is represented by a five-pointed star, tomato by a square with a checkmark, rice by a circle, and Arabidopsis by a triangle.

Based on the phylogenetic and evolutionary analysis of the *RBOH* protein sequences from eggplant, Arabidopsis, tomato, and rice, the *SmRBOHs* are relatively evenly divided into three evolutionary branches, each of which contains *RBOHs* from eggplant, Arabidopsis, tomato, and rice. *RBOHs* clustered in the same branch are more likely to exhibit functional similarities. *SmRBOHA*, *SmRBOHE1*, and *SmRBOHE2* form one evolutionary branch, while *SmRBOHB*, *SmRBOHC*, and *SmRBOHD* form another evolutionary branch. Additionally, *SmRBOHH1* and *SmRBOHH2* constitute a separate evolutionary branch. Except for *SmRBOHA*, which is genetically closest to *AtRBOHF*, the other members of the *SmRBOH* family are genetically closest to *SlRBOH* gene family members. The results suggest that the evolutionary relationship between *SlRBOHs* and *SmRBOHs* is the closest, followed by *AtRBOHs*. Therefore, there might be functional similarities between *SmRBOHs* and tomatoes, as well as Arabidopsis *RBOHs* (Figure 3).

### 3.4. Analysis of SmRBOHs Cis-Acting Elements in Functional Regulation

**Figure 4:**
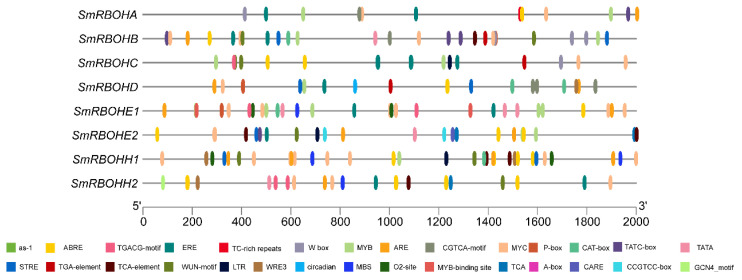
Positions of cis-acting elements on the *SmRBOHs* promoter sequences. The black lines represent the length of the *SmRBOH* promoters (in base pairs, bp), while the rectangles of different colors represent distinct cis-acting elements.

To predict the potential functions of *SmRBOHs*, we extracted a 2000 bp upstream of the initiation codons of the *SmRBOHs* for cis-acting element analysis. The results indicate that *SmRBOHs* contain numerous cis-acting elements, primarily including binding sites for various transcription factors such as MYB, MYC, and W-box; light-responsive elements like Box 4, G-Box, and Sp1; phytohormone-responsive elements such as ABRE, TATC-box, and TGACG-Motif; as well as stress-responsive elements, including STRE, WUN-Motif, and LTR. All *SmRBOH* members possess light-responsive elements (Box 4, G-Box, Sp1, etc.), MYB transcription factor binding sites (MYB), and MYC transcription factor binding sites (MYC). *SmRBOHE1* and *SmRBOHH1* promoter sequences contain the most MYB and MYC binding sites, suggesting that RBOHs may respond to light and act in combination with MYB and MYC. Additionally, W-box is only present in *SmRBOHA*, *SmRBOHB*, and *SmRBOHC*, implying that WRKY transcription factors only bind to a subset of *SmRBOH* members. ABA response elements (ABREs) are found in all *SmRBOH* members, with *SmRBOHH2* containing the highest number of ABREs. Except for *SmRBOHH1*, other members contain ethylene (ETH)-responsive cis-acting elements (EREs), with *SmRBOHC* having the most EREs. With the exception of *SmRBOHE2* and *SmRBOHH1*, other members contain methyl jasmonate (MeJA) response elements (CGTCA-Motif, TGACG-Motif), with *SmRBOHD* containing the highest number of MeJA response elements. Except for *SmRBOHC*, *SmRBOHH1*, and *SmRBOHH2*, cis-acting elements (P-box and TATC-box) related to gibberellin (GA) response were present in other members. In addition, *SmRBOHB*, *SmRBOHE2*, *SmRBOHH1*, and *SmRBOHH2* contain salicylic acid (SA) response elements (TCA-elements). This indicates that *SmRBOHs* can respond to different phytohormones, and *SmRBOHB* contains all the above-mentioned phytohormone response elements, potentially responding to all these phytohormones. Except for *SmRBOHC*, all *SmRBOH* members contain an anaerobic response element (ARE) associated with anaerobic induction. *SmRBOHB*, *SmRBOHD*, *SmRBOHE2*, and *SmRBOHH1* contain stress-responsive element (STRE) cis-acting elements, and apart from *SmRBOHA*, *SmRBOHD*, and *SmRBOHE1*, other *SmRBOHs* members contain mechanical damage response elements (Wun-Motif). *SmRBOHC*, *SmRBOHE2*, and *SmRBOHH1* have low-temperature-responsive LTR elements, while *SmRBOHE1*, *SmRBOHH1*, and *SmRBOHH2* contain drought-responsive MBS elements. Furthermore, some members contain CAT-box, related to meristematic tissue expression, O2-site, related to corn protein metabolism regulation, and elements associated with circadian rhythm regulation. These findings suggest that *SmRBOHs* may have different expression levels and functions under various environmental conditions, potentially participating in the regulation of eggplant growth, development, and multiple stress response pathways (Figure 4).

### 3.5. Expression Analysis of SmRBOHs in Different Tissues

**Figure 5:**
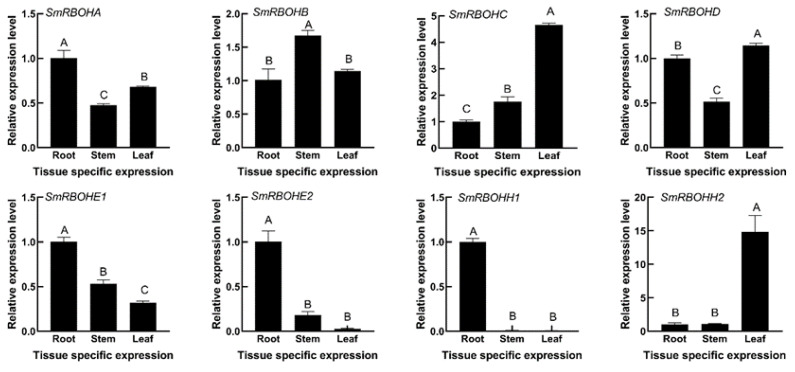
Expression patterns of *SmRBOHs* in roots, stems, and leaves. Based on a one-way analysis of variance and Tukey’s test (*p* < 0.01), the error bar represents the standard deviation, and different uppercase letters indicate highly significant differences in the relative expression levels of *SmRBOHs* among different tissues. The experiment was replicated four times, and *SmActin* was used as the internal reference gene.

We analyzed the relative expression patterns of *SmRBOHs* in the roots, stems, and leaves of eggplant using qRT-PCR. The results revealed differential expression of *SmRBOH* gene members across various tissues. *SmRBOHA*, *SmRBOHE1*, *SmRBOHE2*, and *SmRBOHH1* exhibited the highest expression levels in the roots. *SmRBOHE2* showed relatively lower expression in the leaves, while *SmRBOHH1* displayed reduced expression in both the stems and leaves compared to the roots. *SmRBOHB* showed the highest expression level in the stems. *SmRBOHC* and *SmRBOHD* exhibited the highest expression in the leaves. Therefore, this study suggests that *SmRBOHs* play distinct roles in different tissues of eggplant (Figure 5).

### 3.6. Expression Pattern Analysis of SmRBOHs under Different Stress Conditions

**Figure 6:**
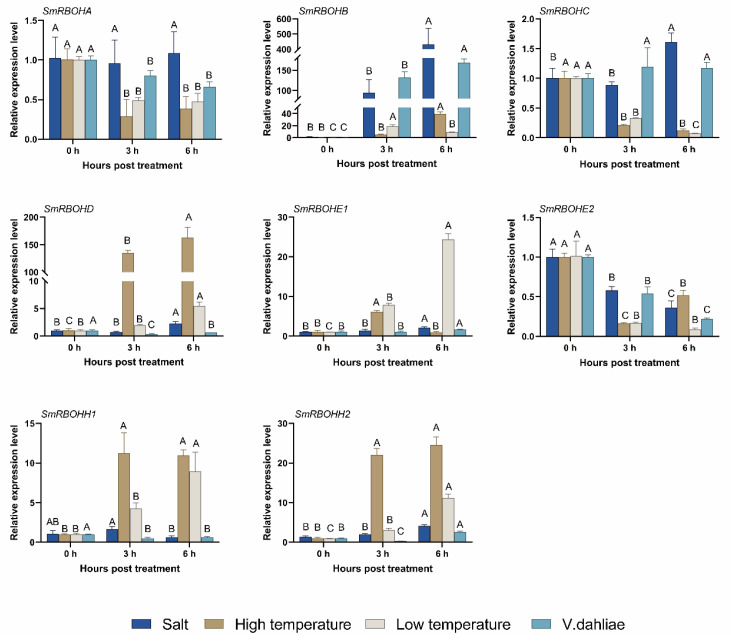
Expression patterns of *SmRBOHs* under salt, high-temperature, low-temperature, and *V. dahliae* inoculation conditions. Based on a one-way analysis of variance and Tukey’s test (*p* < 0.01), the error bar represents the standard deviation, and different capital letters indicate highly significant differences in relative expression levels of *SmRBOHs* at different treatment time points. The experiment was conducted with four replicates, and *SmActin* was used as the reference gene.

To study the response of *SmRBOH* genes under different stress conditions, we subjected 4–6 leaf stage eggplant seedlings to simulated salt stress, temperature stress, and *V. dahliae* treatment. Subsequently, we used qRT-PCR to analyze the relative expression levels of *SmRBOHs* under the aforementioned stress conditions. The results showed differential expression levels of different *SmRBOH* genes under various treatments. Under salt stress, the expression level of *SmRBOHA* showed no significant change, while *SmRBOHE2* and *SmRBOHH1* were downregulated. Conversely, the remaining *SmRBOH* genes showed varying degrees of upregulation. Notably, *SmRBOHB* showed the most substantial upregulation under salt stress, with its expression level increasing over 400-fold at 6 h compared to 0 h. Regarding high-temperature treatment, the expression levels of *SmRBOHA*, *SmRBOHC*, and *SmRBOHE2* were downregulated. *SmRBOHE2* reached its lowest expression level at 3 h and showed a slight upregulation at 6 h, but it was still significantly lower than its expression level at 0 h. *SmRBOHE1* displayed an initial upregulation followed by downregulation, while the other four *SmRBOH* genes showed upregulation under high-temperature conditions, with *SmRBOHD* demonstrating the most significant upregulation. Under low-temperature treatment, the expression levels of *SmRBOHA*, *SmRBOHC*, and *SmRBOHE2* were significantly downregulated, while the expression levels of the remaining *SmRBOH* members were significantly upregulated. Notably, *SmRBOHB* reached its peak expression level at 3 h. During *V. dahliae* inoculation within 6 h, the expression level of *SmRBOHC* showed no significant change, while *SmRBOHB* and *SmRBOHE1* were upregulated in response to *V. dahliae* induction, with *SmRBOHB* showing the most pronounced upregulation (>160-fold). However, the expression of most *SmRBOH* genes decreased after *V. dahliae* inoculation. This study indicates that *SmRBOHs* exhibit different expression patterns under various treatments, suggesting functional diversity among *SmRBOHs*. Additionally, *SmRBOHB* consistently showed significant upregulation under different stress conditions, suggesting its potentially pivotal role in plant response to stress (Figure 6).

### 3.7. Plasma Membrane Localization of SmRBOHs

**Figure 7:**
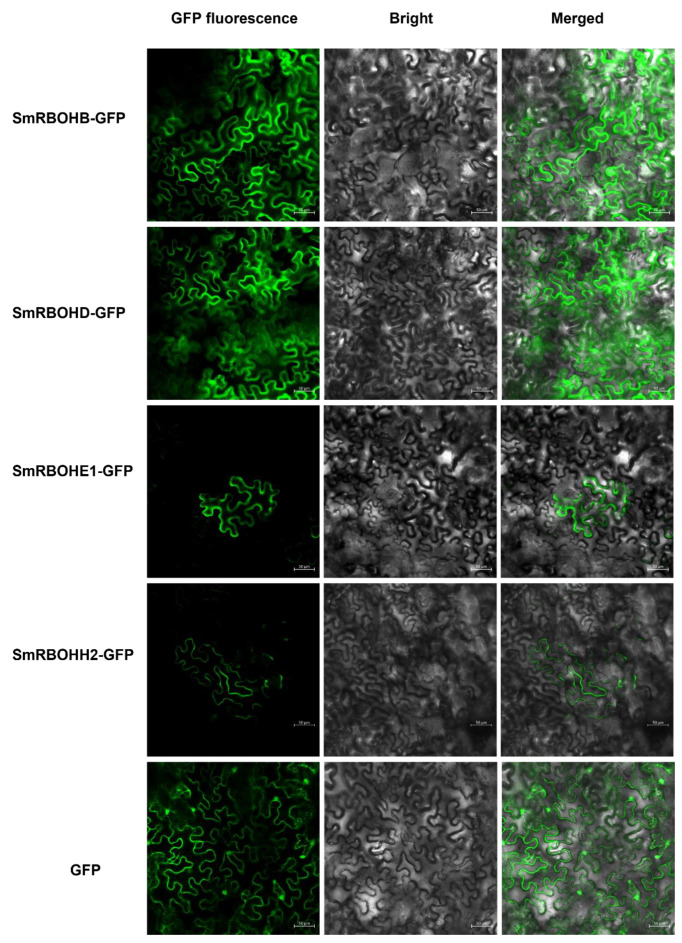
Expression of *SmRBOHs*-GFP fusion proteins in *N. benthamiana* leaves. The *SmRBOHs*-GFP fusion expression vector was transiently expressed in *N. benthamiana* leaves for 48 h, and the expression was observed using laser scanning confocal microscopy. Scale bar = 50 μm.

According to the WOLFSORT online prediction (Section 2.1), *SmRBOHs* are most likely located on the plasma membrane. To verify the above prediction, this study selected several *SmRBOH* members (*SmRBOHB*, *SmRBOHD*, *SmRBOHE1*, and *SmRBOHH2*) that showed a significant upregulation (>20-fold) under stress conditions to construct pBinGFP235S-*SmRBOHs*-GFP expression vectors. Then, we performed transient transformation experiments in the lower epidermal cells of *N. benthamiana* leaves using *Agrobacterium*-mediated transformation. The fluorescence of the above four *SmRBOHs*-GFP fusions was observed on the plasma membrane, indicating that *SmRBOH* proteins primarily localize to the plasma membrane. This finding is consistent with the previously predicted results and suggests that *SmRBOHs* mainly function on the plasma membrane (Figure 7).

## 4. Discussion

ROS, as signaling molecules, play essential roles in mediating plant growth and development, environmental adaptation, and programmed cell death processes. *RBOHs* are crucial enzymes involved in ROS generation. Previous studies have identified ten, nine, and seven members of the *RBOH* gene family in Arabidopsis, rice, and tomato, respectively [7,8]. In this study, we identified eight *RBOH* gene family members in eggplant, which are relatively evenly distributed across seven chromosomes. Studies have indicated that *RBOH* proteins are primarily located on the membrane, catalyzing NADPH in the cytoplasm while transferring electrons to generate ROS [9]. Topological structure analysis of *RBOH* proteins revealed the presence of transmembrane domains in all *RBOHs*, implying their classification as membrane proteins [35]. By utilizing specific antibodies against *RBOHs* to screen different subcellular fractions, only the fractions containing the plasma membrane exhibited positive hybridization bands [36]. In this study, the localization of *SmRBOHs* was predicted to be predominantly on the plasma membrane. We confirmed the accuracy of our predictions through the transient expression of some *SmRBOHs* (*SmRBOHB*, *SmRBOHD*, *SmRBOHE1*, and *SmRBOHH2*) in *N. benthamiana* leaves.

In wheat (*Triticum aestivum* L.), the 46 members of the *NOX* (NADPH oxidase) family are classified into three subgroups based on their conserved domains. The first subgroup consists of typical *TaNOXs*, which possess four conserved domains: NADPH_Ox, Ferric_reduct, FAD_binding_8, and NAD_binding_6. The second subgroup, *TaNOX-likes*, includes members with NADPH_Ox but lack one to two of the other conserved domains. The third subgroup, *FRO* [37], comprises members with the other three conserved domains but lack NADPH_Ox. In this study, the eight *SmRBOHs* all contain the NADPH_Ox domain. SmRBOHE2 is missing the Ferric_reduct domain, while *SmRBOHE1* lacks both the Ferric_reduct and NAD_binding_6 domains. Except for *SmRBOHC*, *SmRBOHE2*, and *SmRBOHH1*, the rest of the *SmRBOHs* are devoid of the FAD_binding_8 domain, which is replaced by the NOX_Duox_like_FAD_NADP domain, and the FAD_binding_8 domain and NOX_Duox_like_FAD_NADP domain serve similar functions [33]. Additionally, SmRBOHs possess 1–2 EF-hand domains (Ef-hand_7 and EFh). These conserved domains have different functions [15]. The NADPH_Ox domain generates ROS, and the C-terminal region containing NAD_binding_6, NOX_Duox_like_FAD_NADP, FAD_binding_8, and PLN02631 superfamily domains play crucial roles in electron transfer and ROS production [33]. The EF-hand domains have a typical helix-loop-helix (HLH) structure and are widely regarded as calcium-binding regions. In the pathway of *RBOH*-mediated ROS production, small GTPases act as regulators, and the N-terminal region is considered a bridge connecting GTPases and RBOHs [38]. *P. tricornutum*’s *RBOHs* lack the EF-hand domain at the N-terminus and do not have homologs that regulate *RBOH* [12], further suggesting the critical role of EF-hand in the interaction between RBOHs and small GTPases [12].

*SmRBOHs* can be classified into three clades in terms of phylogenetic and evolutionary analysis. The evolutionary relationship between *SmRBOHs* and *RBOHs* in tomatoes and Arabidopsis is relatively close. Based on the results of phylogenetic tree analysis, genes that cluster together are more likely to share similar functions and structures. *AtRBOHD* and *AtRBOHF* play an active role under various stresses [23]. Studies have indicated that *TaNOX8* and *TaNOX12* exhibit high sequence similarity to *AtRBOHD* and *AtRBOHF*, respectively, and display similar expression patterns under stress conditions [37]. In this study, the relationship between *SmRBOHD* and *AtRBOHD* is found to be closer in terms of evolutionary relationship. *AtRBOHD* generates ROS under stress conditions by binding to Ca^2+^ and undergoing phosphorylation. *SmRBOHD* is significantly upregulated under temperature stress, especially high-temperature stress, suggesting a potential similarity with *AtRBOHD* in regulating temperature stress responses. On the other hand, *SmRBOHA*, which is evolutionarily closer to *AtRBOHF*, shows significant downregulation under all stresses except for salt stress. This indicates possible functional divergence between *SmRBOHA* and *AtRBOHF*.

Cis-regulatory elements play a crucial role in plant stress and phytohormone responses. In this study, through the analysis of *SmRBOH* promoter regions, numerous stress-related cis-regulatory elements were discovered, such as ARE, Wun-Motif, and STER. These elements are induced by different stressors [39] to regulate the production of ROS, leading to cell death or influencing antioxidant signal transduction in response to various biotic stresses, salt damage [40], drought [41], low-temperature [14], and other abiotic stresses. Previous studies have shown that ROS signaling pathways are closely related to phytohormone regulatory pathways. For example, ABA can induce the expression of the *SlRBOH1* gene, enhance *RBOH* enzyme activity, and improve tomato tolerance [39]. Jasmonic acid compounds (JAs), as important stress signaling molecules, regulate the plant’s antioxidant enzyme system and also participate in the regulation of RBOH activity [42]. ETH and SA regulate the production of ROS dependent on *AtRBOHD* [43]. In this study, all *SmRBOHs* contain the ABA-responsive element (ABRE), and most family members also contain MeJA (CGTCA-Motif, TGACG-Motif)-, ETH (ERE)-, and SA (TCA-element)-responsive elements. The presence of these cis-regulatory elements suggests that the promoter regions of *SmRBOHs* can respond to the regulation of multiple signals.

The expression patterns of genes in different tissues reflect the functional characteristics. In Arabidopsis, 10 *RBOH* genes are distributed across various tissues. Both *AtRBOHD* and *AtRBOHF* are expressed throughout the whole plant, while *AtRBOHH* and *AtRBOHJ* are exclusively present in pollen tubes, and the remaining AtRBOH genes are mainly expressed in the roots [44]. Extensive research has also demonstrated the multifunctionality of *AtRBOHD* and *AtRBOHF*, while *AtRBOHH* and *AtRBOHJ* are involved in regulating pollen tube growth [13,23]. In this study, *SmRBOHs* are expressed in different parts of the plant, showing variations in expression levels. *SmRBOHB* exhibits the highest expression level in the stem, while *SmRBOHC*, *SmRBOHD*, and *SmRBOHH2* show the highest expression in leaves. The remaining *SmRBOH* genes show the highest expression levels in the roots. These results imply that *SmRBOHs* play distinct roles and functions in different plant tissues.

The NADPH oxidases encoded by *RBOHs* on the plasma membrane mediate the production of ROS, which enhances plants’ adaptation to adverse environments. Under abiotic stress, the H_2_O_2_ generated by NADPH oxidases is considered the initial signal of the antioxidant pathway [8,26]. H_2_O_2_ produced by *AtRBOHD* and *AtRBOHF* can trigger an increase in Ca^2+^ levels, thereby regulating the homeostasis of Na^+^/K^+^. Double mutants of *AtRBOHD* and *AtRBOHF* exhibit increased sensitivity to salt stress, with significantly higher Na^+^/K^+^ levels compared to other mutants [26]. In this study, *SmRBOHB* showed a significant upregulation in expression under salt stress, while the expression of other members changed only slightly, indicating that *SmRBOHB* plays a crucial role in the response to salt stress (Figure 6). Changes in temperature can lead to alterations in membrane proteins and membrane lipid composition, affecting membrane permeability and causing cellular damage to plants [45]. Under high-temperature conditions, the expression of *SmRBOHD* was significantly upregulated, and the expression levels of *SmRBOHB*, *SmRBOHH1*, and *SmRBOHH2* also increased notably. This suggests that these genes are involved in eggplant’s heat tolerance process (Figure 6). Under low-temperature conditions, *SmRBOHB* and *SmRBOHE1* showed a substantial increase in expression levels, and compared to the 6 h time point, *SmRBOHB* exhibited even higher expression levels at 3 h. This indicates that *SmRBOHB* plays a role in the early cold tolerance process, while *SmRBOHE1* demonstrates a more sustained effect (Figure 6). Plants generate ROS to trigger immune responses, limiting the invasion of pathogens or enhancing the cell wall to bolster the plant’s defense system under biotic stress. In *A. thaliana*, *AtRBOHD* and *AtRBOHF* respond to various pathogenic infections, with *AtRBOHD* primarily involved in ROS formation and *AtRBOHF* playing a role in cell death [46]. Additionally, the double mutant of *AtRBOHD* and *AtRBOHF* exhibits extreme sensitivity to bacterial AG8, whereas single mutations in *AtRBOH* do not show this effect, suggesting the potential interplay and mutual involvement of *RBOHs* under specific circumstances [47]. *OsRBOHB* expression is induced by rice blast pathogens, and the high concentration of ROS it produces effectively inhibits the hyphal growth and protein secretion of the pathogens, thus restricting further infection by the rice blast fungus [48]. As the causal agent of Verticillium wilt, *V. dahliae* has a wide range of hosts, such as cotton, tomato, and eggplant. The occurrence of Verticillium wilt inflicts irreversible damage to plants. In cotton, GbRboh5/18 participates in the anti-Verticillium wilt process by mediating ROS production [22]. In this study, *SmRBOHB* expression was significantly upregulated upon *V. dahliae* treatment (Figure 6). Based on previous research, we speculate that *SmRBOHB*, induced by *V. dahliae*, generates a substantial amount of ROS, which might be involved in cellular immune responses, restricting the growth of the pathogen. Furthermore, most *SmRBOHs* exhibit downregulated expression under *V. dahliae* infection, and their specific mechanisms of action require further investigation.

Some RBOHs exhibit specificity in their functions, while others display versatility. For instance, *AtRBOHD* and *AtRBOHF* not only play a positive role in response to various stresses such as diseases, salt, and cold stress [23] but also render plants more susceptible to stress, as observed in increased vulnerability to cyst nematode infestation in Arabidopsis [49]. In this study, *SmRBOHB* shows significantly increased expression under different stress conditions, suggesting its multi-functional nature in activating diverse signaling pathways to respond to various stresses.

This research identified *RBOH* genes in eggplant that respond to both biotic and abiotic stresses at the transcriptional level, revealing their involvement in eggplant disease resistance and stress response. This has important implications for molecular breeding and genetic improvement of eggplant. However, a more comprehensive and in-depth experimental investigation is required to elucidate the regulatory mechanisms of *SmRBOHs* under different stress conditions.

## 5. Conclusions

This study identified eight members of the eggplant *RBOH* gene family and analyzed their physicochemical properties, chromosomal localization, Motifs and conserved domains, phylogenetic evolutionary, cis-acting elements, and expression patterns. *SmRBOHs* exhibited diverse expression patterns in different tissues (the roots, stems, and leaves) and various treatments (salt, high-temperature, low-temperature, and *V. dahliae*). Among them, *SmRBOHB* was significantly upregulated under different stress conditions. Additionally, *SmRBOHD*, *SmRBOHE1*, and *SmRBOHH2* also showed significant upregulation (>20-fold) under specific environments, making them potential candidate genes for stress tolerance. Subcellular localization analysis of these four genes revealed that *SmRBOHs* are localized on the plasma membrane. This study provides foundations for further exploring the stress resistance mechanisms of the *SmRBOH* genes in eggplant.

## Figures and Tables

**Table 1:** Basic Information and features of the *SmRBOH* gene family members.

Gene Name	Protein Length/aa	Molecular Weight/KDa	Theoretical Isoelectric Point	Instability Index	Hydrophilia	Subcellular Localization ^1^
*SmRBOHA*	963	109.25	9.2	49.15	−0.237	plas:11, chlo:1, nucl:1, E.R.:1
*SmRBOHB*	866	98.81	8.19	41.93	−0.275	plas:12, nucl:2
*SmRBOHC*	938	105.40	9.13	37.44	−0.296	plas:14
*SmRBOHD*	718	82.14	9.41	38.24	−0.136	plas:9, E.R.:2, nucl:1, cyto: 1, mito: 1
*SmRBOHE1*	843	96.07	8.72	41.98	−0.174	plas:8, E.R.:3, nucl:1, mito:1, pero:1
*SmRBOHE2*	944	106.40	8.79	46.59	−0.153	plas:8, mito:3, chlo:1, nucl:1, pero:1
*SmRBOHH1*	792	90.79	8.83	41	−0.219	plas:13, nucl:1
*SmRBOHH2*	779	89.53	8.95	39.24	−0.142	plas:12, nucl:1, cyto:1

^1^ plas: plasma membrane; ER: endoplasmic reticulum; chlo: chloroplast; nucl: nuclear; cyto: cytoplasmic; pero: peroxisome; mito: mitochondria.

## Data Availability

Not applicable.

## Supplementary Materials

The following supporting information can be downloaded at: https://www.mdpi.com/article/10.3390/genes14091665/s1, Table S1: The *RBOH* gene IDs of eggplant, tomato, rice, and Arabidopsis; Table S2: The protein sequences of the RBOH of eggplant, tomato, rice, and Arabidopsis; Table S3: The primer of *SmRBOHs*-qPCR; Table S4: The primer of *SmRBOHs*.

## References

[1] Kadota Y., Shirasu K., Zipfel C. (2015). Regulation of the NADPH Oxidase *RBOHD* During Plant Immunity. Plant Cell Physiol..

[2] Kora D., Dey A., Pal B., Roy U.K., Dey N., Bhatacharjee T., Bhattacharjee S. (2023). ROS-phytohormone interaction in regulating integrative defense signaling of plant cell. Biocell.

[3] Cheng X., Li G., Manzoor M.A., Wang H., Abdullah M., Su X., Zhang J., Jiang T., Jin Q., Cai Y. (2019). In Silico Genome-Wide Analysis of Respiratory Burst Oxidase Homolog (*RBOH*) Family Genes in Five Fruit-Producing Trees, and Potential Functional Analysis on Lignification of Stone Cells in Chinese White Pear. Cells.

[4] Liu M., Yu H., Ouyang B., Shi C., Demidchik V., Hao Z., Yu M., Shabala S. (2020). NADPH oxidases and the evolution of plant salinity tolerance. Plant Cell Environ..

[5] Sagi M., Davydov O., Orazova S., Yesbergenova Z., Ophir R., Stratmann J.W., Fluhr R. (2004). Plant respiratory burst oxidase homologs impinge on wound responsiveness and development in *Lycopersicon esculentum*. Plant Cell.

[6] Geiszt M., Leto T.L. (2004). The Nox family of NAD(P)H oxidases: Host defense and beyond. J. Biol. Chem..

[7] Kaur G., Pati P.K. (2016). Analysis of cis-acting regulatory elements of Respiratory burst oxidase homolog (*Rboh*) gene families in *Arabidopsis* and rice provides clues for their diverse functions. Comput. Biol. Chem..

[8] Raziq A., Wang Y., Mohi Ud Din A., Sun J., Shu S., Guo S. (2022). A Comprehensive Evaluation of Salt Tolerance in Tomato (*Var. Ailsa Craig*): Responses of Physiological and Transcriptional Changes in RBOH’s and ABA Biosynthesis and Signalling Genes. Int. J. Mol. Sci..

[9] Kobayashi M., Kawakita K., Maeshima M., Doke N., Yoshioka H. (2006). Subcellular localization of Strboh proteins and NADPH-dependent O_2_^−^-generating activity in potato tuber tissues. J. Exp. Bot..

[10] Baker C.J., Deahl K., Domek J., Orlandi E.W. (1998). Oxygen metabolism in plant/bacteria interactions: Effect of DPI on the pseudo-NAD(P)H oxidase activity of peroxidase. Biochem. Biophys. Res. Commun..

[11] Armbrust E.V., Berges J.A., Bowler C., Green B.R., Martinez D., Putnam N.H., Zhou S., Allen A.E., Apt K.E., Bechner M. (2004). The genome of the diatom *Thalassiosira pseudonana*: Ecology, evolution, and metabolism. Science.

[12] Herve C., Tonon T., Collen J., Corre E., Boyen C. (2006). NADPH oxidases in Eukaryotes: Red algae provide new hints. Curr. Genet..

[13] Chapman J.M., Muhlemann J.K., Gayomba S.R., Muday G.K. (2019). RBOH-dependent ROS Synthesis and ROS Scavenging by Plant Specialized Metabolites to Modulate Plant Development and Stress Responses. Chem. Res. Toxicol..

[14] Zhang Y., Li Y., He Y., Hu W., Zhang Y., Wang X., Tang H. (2018). Identification of NADPH oxidase family members associated with cold stress in strawberry. FEBS Open Bio.

[15] Zhang H., Wang X., Yan A., Deng J., Xie Y., Liu S., Liu D., He L., Weng J., Xu J. (2023). Evolutionary Analysis of Respiratory Burst Oxidase Homolog (*RBOH*) Genes in Plants and Characterization of *ZmRBOHs*. Int. J. Mol. Sci..

[16] Li N., Sun L., Zhang L., Song Y., Hu P., Li C., Hao F.S. (2015). *AtrbohD* and *AtrbohF* negatively regulate lateral root development by changing the localized accumulation of superoxide in primary roots of *Arabidopsis*. Planta.

[17] Wang X., Zhang M.M., Wang Y.J., Gao Y.T., Li R., Wang G.F., Li W.Q., Liu W.T., Chen K.M. (2016). The plasma membrane NADPH oxidase *OsRbohA* plays a crucial role in developmental regulation and drought-stress response in rice. Physiol. Plant.

[18] Zhang Y., Zhang Y., Luo L., Lu C., Kong W., Cheng L., Xu X., Liu J. (2022). Genome Wide Identification of Respiratory Burst Oxidase Homolog (*Rboh*) Genes in Citrus sinensis and Functional Analysis of *CsRbohD* in Cold Tolerance. Int. J. Mol. Sci..

[19] Di Q., Li Y., Li S., Shi A., Zhou M., Ren H., Yan Y., He C., Wang J., Sun M. (2022). Photosynthesis Mediated by RBOH-Dependent Signaling is Essential for Cold Stress Memory. Antioxidants.

[20] Kurusu T., Kuchitsu K., Tada Y. (2015). Plant signaling networks involving Ca^2+^ and Rboh/Nox-mediated ROS production under salinity stress. Front. Plant Sci..

[21] Shi Y., Chang Y.L., Wu H.T., Shalmani A., Liu W.T., Li W.Q., Xu J.W., Chen K.M. (2020). *OsRbohB*-mediated ROS production plays a crucial role in drought stress tolerance of rice. Plant Cell Rep..

[22] Chang Y., Li B., Shi Q., Geng R., Geng S., Liu J., Zhang Y., Cai Y. (2020). Comprehensive Analysis of Respiratory Burst Oxidase Homologs (Rboh) Gene Family and Function of GbRboh5/18 on Verticillium Wilt Resistance in *Gossypium barbadense*. Front. Genet..

[23] Torres M.A., Dangl J.L. (2005). Functions of the respiratory burst oxidase in biotic interactions, abiotic stress and development. Curr. Opin. Plant Biol..

[24] Wang R., He F., Ning Y., Wang G.L. (2020). Fine-Tuning of RBOH-Mediated ROS Signaling in Plant Immunity. Trends Plant Sci..

[25] Kapadia C., Datta R., Mahammad S.M., Tomar R.S., Kheni J.K., Ercisli S. (2023). Genome-Wide Identification, Quantification, and Validation of Differentially Expressed miRNAs in Eggplant (*Solanum melongena* L.) Based on Their Response to Ralstonia solanacearum Infection. ACS Omega.

[26] Ben Rejeb K., Benzarti M., Debez A., Bailly C., Savoure A., Abdelly C. (2015). NADPH oxidase-dependent H_2_O_2_ production is required for salt-induced antioxidant defense in *Arabidopsis thaliana*. J. Plant Physiol..

[27] Chen C., Chen H., Zhang Y., Thomas H.R., Frank M.H., He Y., Xia R. (2020). TBtools: An Integrative Toolkit Developed for Interactive Analyses of Big Biological Data. Mol. Plant.

[28] Shen L., Zhao E., Liu R., Yang X. (2022). Transcriptome Analysis of Eggplant under Salt Stress: AP2/ERF Transcription Factor *SmERF1* Acts as a Positive Regulator of Salt Stress. Plants.

[29] Wu L., Gui M., Liu J., Cheng J., Li Z., Bao R., Chen X., Gong Y., Du G. (2023). Comparative Proteomic Analysis of Roots from a Wild Eggplant Species *Solanum sisymbriifolium* in Defense Response to *Verticillium dahliae* Inoculation. Genes.

[30] Ali M., Ahmad H., Hayat S., Ghani M.I., Amin B., Atif M.J., Wali K., Cheng Z. (2021). Application of garlic allelochemicals improves growth and induces defense responses in eggplant (*Solanum melongena*) against *Verticillium dahliae*. Ecotoxicol. Environ. Saf..

[31] Jiang Z., Shen L., He J., Du L., Xia X., Zhang L., Yang X. (2023). Functional Analysis of *SmMYB39* in Salt Stress Tolerance of Eggplant (*Solanum melongena* L.). Horticulturae.

[32] Sagi M., Fluhr R. (2006). Production of reactive oxygen species by plant NADPH oxidases. Plant Physiol..

[33] Lu S., Wang J., Chitsaz F., Derbyshire M.K., Geer R.C., Gonzales N.R., Gwadz M., Hurwitz D.I., Marchler G.H., Song J.S. (2020). CDD/SPARCLE: The conserved domain database in 2020. Nucleic Acids Res..

[34] Zhai L., Sun Q., Gao M., Cheng X., Liao X., Wu T., Zhang X., Xu X., Wang Y., Han Z. (2022). *MxMPK4-1* phosphorylates NADPH oxidase to trigger the *MxMPK6-2*-*MxbHLH104* pathway mediated Fe deficiency responses in apple. Plant Cell Environ..

[35] Kawahara T., Quinn M.T., Lambeth J.D. (2007). Molecular evolution of the reactive oxygen-generating NADPH oxidase (*Nox*/*Duox*) family of enzymes. BMC Evol. Biol..

[36] Simon-Plas F., Elmayan T., Blein J.P. (2002). The plasma membrane oxidase *NtrbohD* is responsible for AOS production in elicited tobacco cells. Plant J..

[37] Hu C.H., Wei X.Y., Yuan B., Yao L.B., Ma T.T., Zhang P.P., Wang X., Wang P.Q., Liu W.T., Li W.Q. (2018). Genome-Wide Identification and Functional Analysis of NADPH Oxidase Family Genes in Wheat during Development and Environmental Stress Responses. Front. Plant Sci..

[38] Wong H.L., Pinontoan R., Hayashi K., Tabata R., Yaeno T., Hasegawa K., Kojima C., Yoshioka H., Iba K., Kawasaki T. (2007). Regulation of rice NADPH oxidase by binding of Rac GTPase to its N-terminal extension. Plant Cell.

[39] Zhou J., Wang J., Li X., Xia X.J., Zhou Y.H., Shi K., Chen Z., Yu J.Q. (2014). H_2_O_2_ mediates the crosstalk of brassinosteroid and abscisic acid in tomato responses to heat and oxidative stresses. J. Exp. Bot..

[40] Ma L., Zhang H., Sun L., Jiao Y., Zhang G., Miao C., Hao F. (2012). NADPH oxidase *AtrbohD* and *AtrbohF* function in ROS-dependent regulation of Na^+^/K^+^ homeostasis in Arabidopsis under salt stress. J. Exp. Bot..

[41] Duan Z.Q., Bai L., Zhao Z.G., Zhang G.P., Cheng F.M., Jiang L.X., Chen K.M. (2009). Drought-stimulated activity of plasma membrane nicotinamide adenine dinucleotide phosphate oxidase and its catalytic properties in rice. J. Integr. Plant Biol..

[42] Alam M.M., Nahar K., Hasanuzzaman M., Fujita M. (2014). Exogenous jasmonic acid modulates the physiology, antioxidant defense and glyoxalase systems in imparting drought stress tolerance in different Brassica species. Plant Biotechnol. Rep..

[43] Liu Y., He C. (2016). Regulation of plant reactive oxygen species (ROS) in stress responses: Learning from *AtRBOHD*. Plant Cell Rep..

[44] Orman-Ligeza B., Parizot B., de Rycke R., Fernandez A., Himschoot E., Van Breusegem F., Bennett M.J., Perilleux C., Beeckman T., Draye X. (2016). RBOH-mediated ROS production facilitates lateral root emergence in *Arabidopsis*. Development.

[45] Luo Q.J., Zhu Z.J., Yang R., Qian F.J., Yan X.J., Chen H.M. (2015). Characterization of a respiratory burst oxidase homologue from *Pyropia haitanensis* with unique molecular phylogeny and rapid stress response. J. Appl. Phycol..

[46] Kwak J.M., Mori I.C., Pei Z.M., Leonhardt N., Torres M.A., Dangl J.L., Bloom R.E., Bodde S., Jones J.D., Schroeder J.I. (2003). NADPH oxidase *AtrbohD* and *AtrbohF* genes function in ROS-dependent ABA signaling in Arabidopsis. EMBO J..

[47] Foley R.C., Gleason C.A., Anderson J.P., Hamann T., Singh K.B. (2013). Genetic and genomic analysis of Rhizoctonia solani interactions with Arabidopsis; evidence of resistance mediated through NADPH oxidases. PLoS ONE.

[48] Li G.B., He J.X., Wu J.L., Wang H., Zhang X., Liu J., Hu X.H., Zhu Y., Shen S., Bai Y.F. (2022). Overproduction of *OsRACK1A*, an effector-targeted scaffold protein promoting *OsRBOHB*-mediated ROS production, confers rice floral resistance to false smut disease without yield penalty. Mol. Plant.

[49] Chopra D., Hasan M.S., Matera C., Chitambo O., Mendy B., Mahlitz S.V., Naz A.A., Szumski S., Janakowski S., Sobczak M. (2021). Plant parasitic cyst nematodes redirect host indole metabolism via NADPH oxidase-mediated ROS to promote infection. New Phytol..

