# Supplementary material for: Genome-Wide Identification and Expression Analysis of Respiratory Burst Oxidase Homolog (RBOH) Gene Family in Eggplant (Solanum melongena L.) under Abiotic and Biotic Stress
Source: Genes (Basel). 2023 Aug 23;14(9):1665. doi: 10.3390/genes14091665 (PMC10531080; doi:10.3390/genes14091665)
Supplement: Supplementary file 1 [file genes-14-01665-s001.zip › supplementary Table S1, S3 and S4.pdf]

Table S1 The *RBOH* genes ID of eggplant, tomato, rice and Arabidopsis

Solanum melongena		***Solanum lycopersicum* [8]**		***Oryza sativa* [7]**		***Arabidopsis thaliana* [7]**	
**Gene name**	**Gene ID**	**Gene name**	**Gene ID**	**Gene name**	Gene ID	Gene name	Gene ID
SmRBOHA	Smechr0802427	*SlRBOH1*	Solyc06g068680	*OsRBOHA*	Os01g0734200	*AtRBOHA*	At5g07390
SmRBOHB	Smechr0101010	*SlRBOH2*	Solyc03g117980	*OsRBOHB*	Os01g0360200	*AtRBOHB*	At1g09090
SmRBOHC	Smechr0303236	*SlRBOH3*	Solyc01g099620	*OsRBOHC*	Os05g0528000	*AtRBOHC*	At5g51060
SmRBOHD	Smechr0602302	*SlRBOH4*	Solyc05g025680	*OsRBOHD*	Os05g0465800	*AtRBOHD*	At5g47910
SmRBOHE1	Smechr0500887	*SlRBOH5*	Solyc07g042460	*OsRBOHE*	Os01g0835500	*AtRBOHE*	At1g19230
SmRBOHE2	Smechr0701288	*SlRBOH6*	Solyc06g075570	*OsRBOHF*	Os08g0453700	*AtRBOHF*	At1g64060
SmRBOHH1	Smechr0602858	*SlRBOH7*	Solyc11g072800	*OsRBOHG*	Os09g0438000	*AtRBOHG*	At4g25090
SmRBOHH2	Smechr1201509			*OsRBOHH*	Os12g0541300	*AtRBOHH*	At5g60010
				OsRBOHI	Os11g0537400	*AtRBOHI*	At4g11230
						AtRBOHJ	At3g45810

Table S3 The primer of *SmRBOHs*-qPCR

Gene name	Forward primer（5′-3′）	Reverse primer（5′-3′）
SmRBOHA-qPCR	TTCCGTCAGTTTTCACAGGA	GAAAAACGACGTCGGAGAAA
SmRBOHB-qPCR	TAAGTCTGGTGCTGCTCGTG	AACCGCAAGTTCATCGAATC
SmRBOHC-qPCR	TAATCACTCGCACCACCATC	TTTTTCCGCCTCGTTTGTT
SmRBOHD-qPCR	CACAAGATTCAAAAGCCGTGT	CTAGTTTTGCCGGTCTTTGC
SmRBOHE1-qPCR	CCTTCATACTGGCAACACGA	GACCAATGCGAGAAGGTGAT
SmRBOHE2-qPCR	TGCCGATTTTCCTCAATGAT	GTGATGGTGTTGGTGCTACG
SmRBOHH1-qPCR	GTACGACCGATGTCCAGGAT	GTGCAACGCCATTTCTTCTT
SmRBOHH2-qPCR	ATCTCACCCAATTCGGTCCT	GGTGAGAATGAAATGGGAACA
SmActin-qPCR	CACTTAGCACCTTCCAGCAGATGT	GTACAACAGCAGACCTGAGTTCACT

Table S4 The primer of *SmRBOHs*

Gene name	Forward primer（5′-3′）	Reverse primer（5′-3′）
SmRBOHB	ATGGAGATCGAAAACACAAGGG	TTAAAAATTTTCTTTATGAAATTCAAACTT
SmRBOHD	ATGATAGGTATGAAAGGAGAATCTCATG	TCAAAAATTTTCTTTATGGAAATCAAA
SmRBOHE1	ATGAGAAAAGTGTCATCTCCTAGGAGC	TCAGAAGTACTCCTTGTGGAACTCG
SmRBOHH2	ATGGCGGAATACCCTTTCGA	CTAGAAGTTCTCTTTGTGGAAGTTGAA
